# Bicuspid aortic valve repair—current techniques, outcomes, challenges, and future perspectives

**DOI:** 10.3389/fcvm.2023.1295146

**Published:** 2024-01-03

**Authors:** Haiyu Zhang

**Affiliations:** ^1^ School of Biological and Behavioural Sciences, Queen Mary University of London, London, United Kingdom; ^2^ Department of Cardiothoracic Surgery, The First Affiliated Hospital of Nanchang University, Nanchang, Jiangxi, China

**Keywords:** bicuspid aortic valve repair, aortic surgery, aortopathy, aortic regurgitation, congenital valve disease

## Abstract

Bicuspid aortic valve (BAV) is a common congenital heart condition that can lead to some valve-related complications, such as aortic stenosis and/or regurgitation, and is often associated with aortic root dilation. With the development and refinement of BAV repair techniques over the past three decades, surgical repair of BAV has emerged as an effective treatment option, offering symptomatic relief and improved outcomes. This review aims to summarize the current techniques, outcomes, and challenges of BAV repair, and to provide potential future perspectives in the field.

## 1. Introduction

Bicuspid aortic valve (BAV) is the most common congenital heart condition, occurring in 0.7%–1.4% of the general population (2–3:1 male predominance) (1). Most BAV patients are asymptomatic in early life but develop some complications over time, including aortic stenosis, aortic dilation, aortic regurgitation, coarctation, endocarditis, and dissection (2). Currently, BAV repair and aortic valve replacement (AVR) are the two main surgical options to treat BAV disease. AVR with biological tissue valve or mechanical valve is a conventional approach. However, a biological tissue valve only lasts about 10–18 years due to degeneration, and a mechanical valve requires lifelong anticoagulation. With the development and improvement of BAV repair techniques over the past three decades, BAV repair have achieved excellent outcomes. Many patients receive BAV repair when feasible to avoid the limitations of classical AVR. A propensity score analysis study reported that aortic valve repair had similar operative mortality (2% vs. 5%), better overall 9-year survival (87% vs. 60%), and a slightly higher reoperation rate (8% vs. 2%) compared to AVR (3). Although direct comparisons between BAV repair and AVR are currently lacking, BAV repair is expected to emerge as a more attractive procedure for treating BAV disease.

## 2. BAV classification

Understanding the classification of BAV is crucial for surgeons to choose appropriate BAV repair procedures for different BAV condition. BAV phenotypes and BAV-associated aortopathy have been described diversely by some researchers, while a complete standard classification system was lacking for a long time before the publishment of the international consensus statement in 2021 (4).

The statement summarized three types of bicuspid valves: fused BAV, 2-sinus BAV, and partial-fusion (forme fruste) BAV. The most common type is the fused BAV, with two of the three cusps fused together within three aortic sinuses, which is specifically classified into right-left cusp fusion, right non-cusp fusion, left non-cusp fusion and indeterminate phenotypes. The 2-sinus BAV has two roughly identical cusps and two aortic sinuses, including latero-lateral and antero-posterior phenotypes. The partial-fusion BAV phenotype is characterized by small (less than 50%) fusion between two cusps at the base of one commissure, forming a “mini-raphe” (4).

In addition, three categories of BAV-associated aortopathy were identified in the statement: (1) The ascending phenotype with preferential dilation at the tubular ascending aorta; (2) The root phenotype that preferentially dilates at the root; (3) The extended phenotype with extended dilation of ascending or root phenotype to adjacent segments (4).

## 3. BAV repair techniques

In 1983, Alain Carpentier initially concluded specific techniques for aortic valve repair, including commissurotomy and cusp shaving for restricted cusp motion, circular suture for annular dilation, and triangular resection for cusp prolapse. He suggested that about 80% of congenital aortic valve malformation cases were feasible. At that time, the repair techniques were recommended only as an alternative for valve replacement in children due to insufficient clinical experience, and calcified aortic valves were not applicable (5). Over the past few decades, with in-depth analyses of BAV repair results, several factors that can influence the repair results have been identified, such as age, aortic root diameter, effective height, commissural orientation, the use of a pericardial patch, etc. (6). The recognition of the influencing factors has led to the advancement of specific techniques and surgical strategies.

### 3.1. Considering aortic root

Dilation at the aortic root is common in BAV patients. Studies have found that the degree of aortic root dilation is correlated with the degree of aortic regurgitation (AR), and dimensions of the aortic annulus and the sino-tubular junction (STJ) were independent predictors of AR progression for BAV patients (6, 7). Moreover, patients with constant dilated aortic root after BAV repair often require reoperation due to recurrent AR (6, 8). With the aim of normalizing the aortic root in BAV repair, various approaches have been proposed and applied.

In early period, annuloplasty with subcommissural plication sutures first proposed by Cabrol et al. was used by many BAV repair groups to stabilize the annulus (9). However, it has been abandoned by most surgeons because it does not provide durable annular stabilization consistently and is associated with repair failure (6, 10).

**Figure 1:**
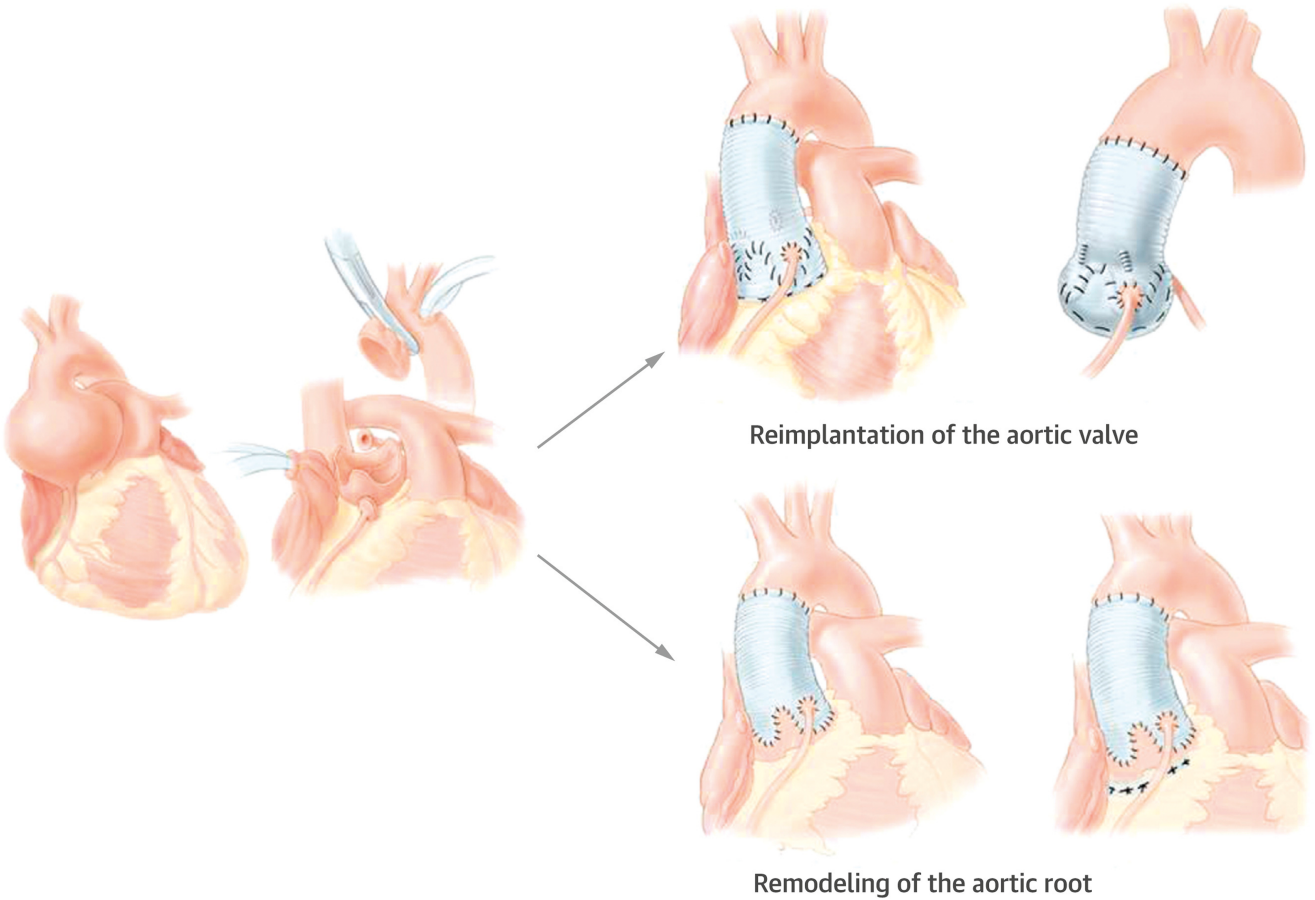
Valve-sparing aortic procedures: the David reimplantation procedure reimplants the aortic valve within a Dacron graft (12). The Yacoub remodeling procedure creates three artificial sinuses of Valsalva with a Dacron graft (11). Reprinted from David (13), Copyright 2016, with permission from Elsevier.

The Yacoub remodeling procedure and the David reimplantation procedure are two types of valve-sparing aortic root surgery (Figure 1). The Yacoub procedure reconstitutes the aortic root and creates three artificial sinuses of Valsalva with a tubular Dacron graft which is scalloped at one end (11). Ongoing dilation of the ventriculo-aortic junction (VAJ) is a common cause of repair failure due to the lack of VAJ stabilization in this procedure. Therefore, Lansac et al. proposed to apply subvalvular external aortic prosthetic ring annuloplasty in the Yacoub remodeling procedure and showed improved results (8). The David procedure reimplants the aortic valve within a Dacron graft. Both the VAJ and the STJ are stabilized while the sinuses of Valsalva are abolished (12). Kerchove et al. showed that the David procedure improved valve mobility and repair durability compared with subcommissual annuloplasty or isolated cusp repair (14).

With the experience of adding external prosthetic ring annuloplasty in the Yacoub procedure, Lansac et al. then applied the annuloplasty to cusp repair in AR only patients (sinuses of Valsalva <40 mm) (15). While Schneider and colleagues thought that external ring annuloplasty could cause aortic root distortion, as relevant height discrepancy between the aortic annulus and the VAJ were found in 20%–30% of the BAV patients. Given the anatomical variations, they modified the suture annuloplasty initially proposed by Taylor et al. and applied that at the basal level of the root (16–18). Later analysis of the midterm results showed that suture annuloplasty significantly improved BAV repair stability compared to isolated BAV repair, and using expanded polytetrafluorethylene (PTFE) had better repair durability and minimal local complications than using braided polyester (18).

**Figure 2:**
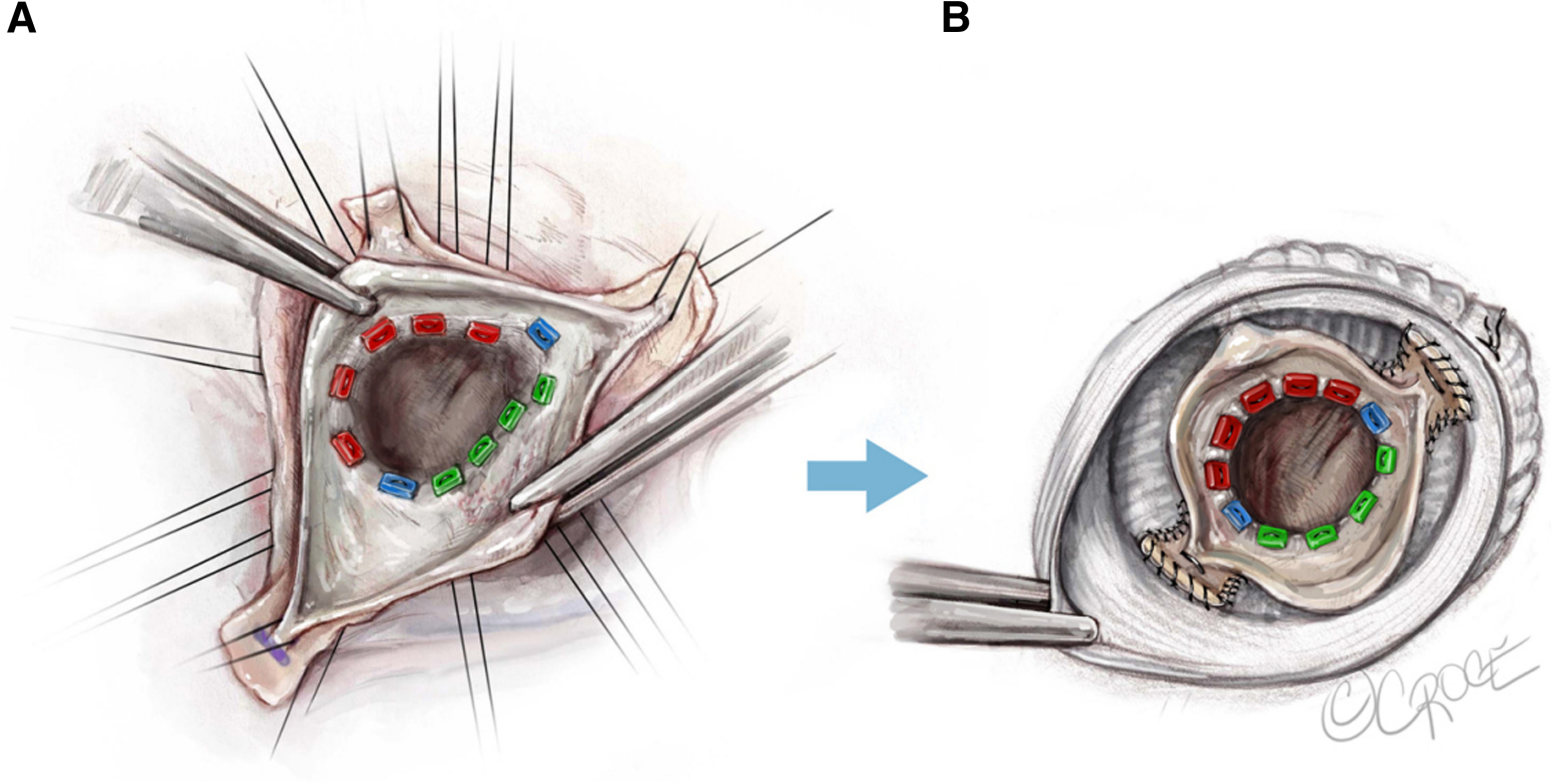
El Khoury technique, a modified David procedure with selective annuloplasty and reimplantation of commissures at 180° (19). Used with permission of AME Publishing Company, from Jahanyar (19); permission conveyed through Copyright Clearance Center, Inc.

The 180°-Reimplantation technique (El Khoury technique) is a modification of the David procedure. It uses a selective annuloplasty to create a symmetric valve and stabilizes the functional aortic annulus through reimplantation of the commissure at 180° at the level of the virtual basal ring and the STJ (Figure 2). Jahanyar et al. reported excellent long-term results and concluded that the technique is suitable for most BAVs except for patients with connective tissue disorders, while some very asymmetric and tricuspid aortic valve-like phenotypes can be better repaired by tricuspidization (19).

### 3.2. Considering leaflet prolapse

In the past, there was no efficient way to quantify cusp geometry until Schäfers et al. introduced the concept of “effective height” and designed a specific caliper for the identification of prolapsing leaflet and the evaluation of prolapse correction outcomes (20). However, the measurement of effective height is only applied in nonfused cusps and as reference for fused cusps because the geometric determinants of effective height are variable in fused cusps, especially the aortic insertion (21). The normal range of effective height in a bicuspid valve is 9–10 mm (22). Nonfused cusp with an effective height less than 9 mm is considered as prolapse and require surgical correction (16, 19). Currently, free margin plication and resuspension are common techniques used for correcting prolapse. The free margin plication technique is highly effective in addressing minor discrepancy in leaflet lengths, while the free margin resuspension technique is particularly useful for prolapsed cusps with fragile free margin or fenestration (23).

### 3.3. Considering commissural orientation

Commissural angle has great influence on BAV repair durability. A commissural orientation of 160°–180° is associated with good repair durability (6). Several techniques have been applied to reposition commissures. Kari et al. analysed the outcomes of BAV patients undergone valve-sparing aortic root replacement and found that commissural orientation increased in patients with no or single raphe (24). Schneider et al. proposed a method to modify commissural orientation by plicating the fused sinuses and showed improved repair stability (18). Later, Urbanski and colleagues developed a modified remodeling technique to achieve symmetric 180° commissural orientation by enlarging the unfused sinus with a patch and narrowing the aortic root wall on the opposite side, which is simple and effective (25).

### 3.4. Considering the use of pericardial patch

Pericardial patch has been widely used in aortic valve repair, especially for the augmentation of retracted cusps, cusp reconstruction after triangular resection in the presence of severe calcification, and the closure of endocarditic perforations (21). Karliova et al. reported that the use of pericardial patch in BAV repair increased the reoperation rate regardless of cusp pathology and repair technique, while reliable long-term competence of reconstructed BAVs following pericardial patch augmentation was achieved by Doss et al. with the principle of retaining the bicuspid morphology of the incompetent valve, enhancing the free edge of the fused leaflet with a strip of glutaraldehyde-fixed pericardium, creating large coaptation, and restoring the belly shape of the fused leaflet for optimal stress distribution (26–28). Therefore, when considering the use of pericardial patch in BAV repair, it is important to seriously evaluate valve morphology and cusp pathology, and formulate an appropriate surgical plan to achieve good results.

## 4. BAV repair outcomes

With the development and refinement of BAV repair techniques over the past few decades, bicuspid aortic valve repair has yielded promising outcomes. Svensson et al. evaluated the long-term outcomes of BAV repair with a mean follow-up of 9 years. A total of 728 patients underwent BAV repair at Cleveland Clinic with an average age of 42 were included. The results indicated that BAV repair is a safe and durable procedure, with low rates of hospital mortality (0.41%) and stroke (0.27%). The long-term survival rate at 10 years was reported to be 94%. The risk of reoperation decreased significantly, at a rate of approximately 2.6% per year, over a period of up to 15 years. The primary reasons for reoperation were identified as cusp prolapse (38%), aortic stenosis or regurgitation (17%), and aortic regurgitation resulting from a root aneurysm (15%) (29). In a more recent study, Arnaoutakis et al. conducted a pooled analysis of the results from 26 studies on BAV repair, which further supported the acceptable long-term outcomes achieved through this procedure (30). However, several crucial factors should be considered in order to achieve satisfactory outcomes. First, surgical expertise and experience are crucial. Experienced surgeons who have performed large amounts of BAV repair procedures tend to have better outcomes. Second, meticulous patient selection is essential for selecting the most appropriate surgical strategy, including patient general characteristics, valve morphology, valve function, and associated aortic pathology. Also, postoperative management and regular follow-up play important roles in monitoring valve function and detecting any potential complications early.

## 5. Challenges

Despite the advancements in BAV repair techniques, there are still some challenges and issues need to be addressed. First, a standardized approach to patient selection and surgical techniques is lacking, leading to variations in outcomes across different centers. Establishing consensus guidelines and protocols can help standardize surgical practice and improve outcomes. Second, although the positive outcomes of BAV repair have been reported in existing literatures, most studies are retrospective in nature, with varying sample sizes and follow-up durations. Prospective studies comparing different surgical techniques and approaches with consistent control of confounding predictors are necessary to provide more robust evidence. Moreover, studies assessing patients' quality of life and functional outcomes are insufficient. Dedicated studies with larger cohorts and standardized outcome assessment are necessary to further enhance our understanding about the benifits of BAV repair.

## 6. Future perspectives

### 6.1. Minimally invasive approaches

In recent years, minimally invasive approaches have been applied broadly in cardiac surgeries due to potential advantages of reduced surgical trauma and rapid recovery (31–33). The early post-operative results of valve-sparing David procedure via minimally invasive access have been reported to be comparable to those via full sternotomy (31). In the future, minimally invasive approaches are expected to applied to a variety of BAV repair strategies, and adequate follow-up studies are needed to assess the effectiveness.

### 6.2. Emerging technologies

Emerging technologies are expected to enhance the precision and effectiveness of BAV repair. The application of three-dimensional (3D) printing and virtual surgical planning, especially in uncommon and high-risk situation, can help surgeons create patient-specific models and simulate complex procedures preoperatively, thereby improving surgical accuracy and reducing operative time (34). Additionally, the use of advanced imaging, such as transesophageal echocardiography (TEE) and four-dimensional cardiovascular magnetic resonance flow imaging (4D Flow CMR), can provide detailed and accurate evaluation of BAVs, leading to better surgical planning and outcomes (35). Further studies are needed to validate the utility of the technologies in BAV repair and their impact on the outcomes.

### 6.3. BAV-related genetics

BAV has been demonstrated to have a significant genetic basis (36). Some genes associated with BAV development have been identified, such as NOTCH1, SMAD6, GATA4, GATA5, GATA6, ROBO4, etc. (37–42). However, the exact pathogenesis of BAV is not fully understood. Understanding the genetic and epigenetic underpinnings of BAV can provide insights into disease mechanisms, guide the identification of potential therapeutic targets, promote the development of novel personalized management strategies and achieve individual risk stratification with the help of genetic testing to avoid unnecessary interventions for low-risk patients and prevent potentially fatal complications early for high-risk patients (43). Therefore, it is crucial to further discover and validate BAV-related clinical and genetic markers. Furthermore, their impact on surgical decision-making and clinical outcomes should be evaluated.

### 6.4. Multidisciplinary collaboration

Advancing the field of BAV repair also requires in-depth multidisciplinary effort. Collaboration between cardiac surgeons, cardiologists, geneticists, and imaging specialists can provide more comprehensive patient evaluation, optimal surgical planning, and sufficient follow-up. Moreover, valuable data regarding surgical outcomes, complications, and long-term durability can be shared through international databases. These collaborative efforts help identify areas for improvement, refine surgical techniques, and provide directions for future research.

## 7. Conclusion

BAV repair techniques have evolved significantly over the past three decades. Continued research and advancements in surgical techniques, emerging technologies, BAV-related genetics, and collaborative research are expected to further improve the outcomes of BAV repair, ultimately activating the full potential of BAV repair and provide personalized and effective treatment for individuals with BAV.
